# Anthropometric measurements in childhood and prediction of cardiovascular risk factors in adulthood: Kaunas cardiovascular risk cohort study

**DOI:** 10.1186/s12889-015-1528-5

**Published:** 2015-03-04

**Authors:** Janina Petkeviciene, Jurate Klumbiene, Vilma Kriaucioniene, Asta Raskiliene, Edita Sakyte, Indre Ceponiene

**Affiliations:** Faculty of Public Health, Medical Academy, Lithuanian University of Health Sciences, Siaures av. 57, Kaunas, Lithuania; Department of Cardiology, Medical Academy, Lithuanian University of Health Sciences, Eiveniu 2, Kaunas, Lithuania

**Keywords:** Body mass index, Skinfold thickness, Cardiovascular risk factors, Longitudinal cohort study

## Abstract

**Background:**

This study aimed to examine the associations between anthropometric measurements in childhood and adulthood as well as the effect of childhood body mass index (BMI) and skinfold thickness in the prediction of adult cardiovascular risk factors.

**Methods:**

The Study subjects were participants of the Kaunas Cardiovascular Risk Cohort study. They were 12–13 years old at the time of the baseline survey (1977) and 48–49 years old in the 35-year follow-up survey (2012, n = 506). In childhood, height, weight, subscapular and triceps skinfold thickness measurements were taken. In 2012, health examination involved measurements of blood pressure (BP), BMI, waist circumference, glucose, lipids, and high-sensitivity C-reactive protein (CRP) levels. Logistic regression models were fitted to assess the associations of childhood BMI and skinfold thicknesses as well as BMI gain with cardiovascular risk factors in middle age. All logistic regression models were adjusted for sex, physical activity level, alcohol consumption, smoking and family history of obesity.

**Results:**

Over 35 years of follow-up, BMI gain was greater in men than in women. Anthropometric measurements in childhood significantly correlated with values measured in adulthood. The highest correlation coefficients were defined for weight and BMI measurements (in girls r = 0.56 and r = 0.51 respectively; in boys r = 0.45 and r = 0.41 respectively, P < 0.001). Mean values of change in BMI were similar in all quintiles of childhood BMI; however, prevalence of adult obesity increased considerably with increasing quintiles. The risk of adult obesity, metabolic syndrome, hyperglycaemia or type 2 diabetes, and elevated level of high-sensitivity CRP increased with a rise in childhood BMI and skinfold thicknesses, irrespectively of BMI gain from childhood to adulthood. No relationship was found between childhood anthropometric measurements and arterial hypertension, raised level of triglycerides or reduced level of HDL cholesterol. Gain in BMI from childhood to adulthood was associated with increased odds of all above-mentioned risk factors independently of childhood BMI.

**Conclusions:**

Risk of metabolic syndrome, hyperglycaemia and diabetes, and elevated high-sensitivity CRP may be affected by childhood BMI and skinfold thickness, while risk of hypertension, raised triglycerides and reduced HDL cholesterol is associated more strongly with BMI gain from childhood to adulthood.

## Background

The growing prevalence of overweight and obesity in children is a serious public health concern. Worldwide, proportion of overweight and obese children rose by 47.1% between 1980 and 2013 [1]. In 2013, 23.8% of boys and 22.6% of girls were overweight or obese in developed countries. In Lithuania, the proportion of overweight boys (13%) and overweight girls (8%) aged 13 years was one of the lowest among all 39 countries, participating in Health Behaviour in School-aged Children study (2009/2010) [2]. However, recent studies provided evidence that prevalence of overweight is increasing in the country, particularly among primary-school children [3].

Overweight children are known to be more likely to become obese adults than children with normal weight [4-7]. There is substantial evidence that obesity in adulthood is strongly related to cardiovascular risk [8,9]. Data of prospective studies showed that each increase of 5 kg/m^2^ in body mass index (BMI) was associated with 40% higher cardiovascular mortality [9]. This association is explained by the adverse cardiovascular risk profile in obese individuals characterized by high blood pressure (BP), dyslipidaemia, hyperglycaemia, elevated level of C-reactive protein (CRP) and higher level of other biomarkers of cardiovascular risk [10,11]. However, the contribution of childhood obesity to cardiovascular risk in adulthood is not clearly defined. A meta-analysis of prospective studies showed that for children 7 to 18 years old a 1 kg/m^2^ increase in BMI was associated with a 5% increase in the risk of coronary heart disease (CHD) in adults [12]. Nevertheless, there was a considerable statistical heterogeneity in the relative risk of outcome between studies. In a systematic review, Lloyd et al. found little evidence to suggest that childhood obesity is an independent cardiovascular risk factor [13]. Several studies demonstrated that prediction of cardiovascular risk depended on obesity pattern. Permanent obesity through childhood to adulthood had greater effect on cardiovascular risk than adult obesity only [14,15]. In contrast, obese children who became non-obese by adulthood had the same risk of outcomes as individuals who never experienced obesity [14]. It is still unclear whether the observed associations between childhood obesity and adult cardiovascular risk reflect the tracking of BMI from childhood to adulthood or whether childhood adiposity has an independent effect on risk of adverse health outcomes, irrespectively of the degree of adult adiposity.

From a public health perspective, early prevention of cardiovascular diseases (CVD) should be given high priority in Lithuania, a post-communist transition country with a very high mortality from CVD and a growing prevalence of overweight in children [16]. For early prevention, it is important to know the links between childhood health indicators and CVD risk in adulthood.

In the current study, the associations between anthropometric measurements in childhood and adulthood as well as the effect of childhood BMI and skinfold thickness in the prediction of adult cardiovascular risk factors were examined using the data from Kaunas (Lithuania) 35-year follow-up cohort study.

## Methods

### Study design and sample

The Kaunas Cardiovascular Risk Cohort study was initiated in 1977, when a random sample of Kaunas schoolchildren born in 1964 (n = 1082) was examined within the framework of the International Study of Juvenile Hypertension [17]. Fifteen secondary schools of Kaunas city were randomly selected to participate in the study. In each school all 6th year schoolchildren were examined. In 2012, a follow-up survey of the cohort was carried-out. Over 35-year period, 91 individuals (8.4%) died, 103 subjects (9.5%) emigrated from Lithuania, 4 (0.4%) individuals were seriously ill, and the addresses of 90 (8.3%) subjects were not available in the National Population Register; therefore, the eligible sample consisted of 794 subjects. A total of 507 subjects (63.9% of eligible sample), aged 48–49 years, participated in the last follow-up survey. For one disabled subject (sitting in a wheelchair) not all anthropometric measurements were taken; therefore he was excluded from the analysis.

The study protocol was approved by the Lithuanian Bioethics Committee. Written consent on behalf of the children enrolled in the first survey (1977) was obtained from parents or guardians. Written informed consent for the participation in the follow-up survey (2012) was obtained from all participants.

### Measurements in childhood

Anthropometric measurements were obtained by trained examiners according to a standardized protocol. The height of participants, without shoes, was measured to the nearest centimetre with a stadiometer. The body weight of participants, wearing light indoor clothing and no shoes, was measured to the nearest 0.1 kg with standardized medical scales. BMI was calculated as weight divided by height squared (kg/m^2^). In childhood, overweight and obesity were defined using age and sex specific cut-off points for BMI recommended by the International Obesity Task Force (IOTF) [18].

The triceps and subscapular skinfold thicknesses were measured two times to the nearest 1.0 mm with a Harpenden calliper and the mean values were used in the analyses. The triceps skinfold was taken on the mid-line of the posterior surface of the arm (over the triceps muscle) at the level of the mid-point between the acromiale and the radiale. The subscapular skinfold was measured just below the angle of the scapula. The sum of the triceps and subscapular skinfold thicknesses was used in the logistic regression analysis.

BP was measured from the right brachial artery with a standard mercury sphygmomanometer in the sitting position after 5 minutes of rest. BP measurements were performed to the nearest 2 mmHg. The first Korotkoff phase was used to determine systolic BP, and the fifth phase was used to determine diastolic BP. Three consecutive BP measurements were taken. The average of these three measurements was used in the analysis.

### Measurements in adulthood

Height and weight were measured following the same methodology as in childhood. Overweight in adulthood was defined as BMI of 25–29.9 kg/m^2^, and obesity as BMI equal to or higher than 30 kg/m^2^.

Waist circumference was measured at the mid-point between the lower margin of the last palpable rib and the top of the iliac crest using a stretch-resistant tape, to the nearest 0.5 cm.

BP measurements were obtained following the same methodology as in childhood. Hypertension in adulthood was defined as systolic BP ≥ 140 mmHg and/or diastolic BP ≥ 90 mmHg or BP < 140/90 mmHg using antihypertensive medication for the last two weeks before examination.

Blood samples for lipids, glucose, and high-sensitivity CRP measurements were taken in the morning after fasting at least 12 hours. All measurements were performed in a certified laboratory on an automatic analyser Cobas Integra 400 plus. Serum lipid and glucose levels were determined using conventional enzymatic methods. Hexokinase was used for measurement of glucose level. High-sensitivity CRP level was assessed using immunoturbidimetric method.

Metabolic syndrome was determined according to criteria of International Diabetes Federation: central obesity defined as waist circumference ≥94 cm for men and ≥80 cm for women plus any two of the following four factors: raised triglycerides level (≥1.7 mmol/L); reduced high-density lipoprotein (HDL) cholesterol level (for men <1.03 mmol/L and for women <1.29 mmol/L); raised blood pressure (systolic BP ≥ 130 mm Hg or diastolic BP ≥ 85 mm Hg, or treatment of previously diagnosed hypertension); raised fasting plasma glucose (≥5.6 mmol/L or previously diagnosed type 2 diabetes) [19]. Participants were classified as having type 2 diabetes mellitus if they had received a diagnosis from a physician and reported the use of glucose-lowering medication. Type 2 diabetes was diagnosed only for 15 individuals; therefore, this outcome was analysed together with hyperglycaemia.

Dyslipidaemias and hyperglycaemia were defined using the same criteria as for metabolic syndrome. The data on the use of lipid lowering medications were collected. Only 37 participants (7.3%) used such medications. All of them had elevated levels of total and LDL cholesterol at the time of health examination; therefore they were included in the group of participants having hypercholesterolaemia or high level of LDL cholesterol. High-sensitivity CRP level equal to or higher than 3 mg/L was considered as elevated [20].

A standard questionnaire was applied to obtain data on alcohol consumption. The questionnaire contained questions about type and frequency of alcohol consumption and amount of alcohol consumed at one occasion. The amount of alcohol consumed at one occasion was recalculated into standard alcohol units (SAUs) using the following formula: SAUs = amount (in litres) × strength of alcoholic drink (beer – 5%, wine – 12%, strong alcohol – 40%). One SAU equals to 10 g of ethanol. The amount of SAUs consumed during a month was calculated. Risky alcohol consumption was defined as drinking of 56 SAUs or more per month for men and drinking of 28 SAUS or more per month for women. Information on cigarette smoking was collected using a standard questionnaire. The questionnaire contained questions about frequency and intensity of smoking. Participants were divided in daily smokers and others. Physical activity was assessed using the International Physical Activity Questionnaire (IPAQ) [21]. Physical activity of less than 600 MET-min/week was considered as low. Family history of obesity was defined as positive if a participant reported that at least one of the parents, brothers or sisters was obese.

### Statistical analysis

Categorical variables were expressed as percentages and tested by the *χ*^2^ test. The normality of distribution of continuous variables was tested by Kolmogorov-Smirnov test. Means and standard deviations (SD) were presented for the normally distributed continuous variables while median and interquartile range were calculated for the distributions that did not meet the criteria of normality. Student *t* test and analysis of variance were used to compare the mean values of normally distributed variables and Mann–Whitney test was applied for the comparison of non-normal distributions. Spearman correlation coefficients were calculated to determine the associations between anthropometric measurements in childhood and adulthood. Mean BMI gain from childhood to adulthood and adult obesity prevalence were calculated in quintiles of childhood BMI.

Logistic regression analysis was performed to determine the associations of childhood BMI and the sum of the skinfold thicknesses with adult cardiovascular risk factors without and with adjustment for the change in BMI from childhood to adulthood. A separate logistic regression model was calculated for every risk factor. All logistic regression models were adjusted for sex, physical activity level, alcohol consumption, smoking and family history of obesity. Modification of the main effect of childhood BMI by sex was assessed by the inclusion of the interaction term in all models. Interaction terms were not statistically significant (p > 0.05), therefore the results were presented for both genders. All statistical analyses were performed using statistical software package SPSS version 20.0 for Windows. P values of less than 0.05 were considered to be statistically significant.

## Results

The baseline characteristics in childhood, including anthropometric measurements, were compared between those who participated (n = 506) and those who did not participate (n = 576) in the 2012 follow-up survey. No statistically significant differences of analysed variables were found between the groups (data are not shown).

The characteristics of the study participants in the first survey (1977) and in the last survey (2012) are presented in Table 1. In childhood, the mean values of all anthropometric measurements of girls were higher compared with boys. Despite this fact, no sex differences in the prevalence of overweight and obesity defined using IOTF age- and sex-specific criteria were observed. Almost every tenth child (11.3% of boys and 12.7% of girls) was overweight or obese. Over 35 years of follow-up, an increase in height and weight, also BMI gain was greater in men than in women. In adulthood, the prevalence of overweight reached 43.9% in men and 35.0% in women (P = 0.01). Proportion of obese men and women (25.2% and 21.4%, respectively) did not differ significantly. The mean value of waist circumference was higher in adult men than in women.

**Table 1:** **Characteristics of the study population in childhood and adulthood**

**Characteristic**	**Men**	**Women**	**P value**
	**n = 230**	**n = 276**	
**Childhood (12–13 years)**			
Height, cm*	155.2 (7.8)	158.4 (6.9)	<0.001
Weight, kg**	42.9 (13.2)	47.1 (13.0)	<0.001
BMI, kg/m^2^**	17.8 (3.4)	18.6 (3.3)	0.003
Triceps skinfold thickness, mm**	10.2 (5.1)	13.7 (6.4)	<0.001
Subscapular skinfold thickness, mm**	5.5 (2.4)	7.3 (3.8)	<0.001
Overweight/obesity prevalence, %	11.3	12.7	0.623
Obesity prevalence, %	1.7	2.5	0.528
**Adulthood (48–49 years)**			
Height, cm*	179.3 (6.3)	167.1 (6.0)	<0.001
Weight, kg**	86.5 (20.0)	70.4 (20.1)	<0.001
BMI, kg/m^2^**	26.9 (5.6)	25.6 (6.6)	0.008
Waist circumference, cm*	97.2 (12.5)	85.0 (13.5)	<0.001
Overweight prevalence, %	43.9	35.0	0.01
Obesity prevalence, %	25.2	21.4	0.341
Change in BMI from childhood to adulthood, kg/m^2^*	9.1 (4.6)	7.6 (4.4)	<0.001
Hypertension, %	60.2	30.8	<0.001
Metabolic syndrome, %	33.6	18.5	<0.001
Hyperglycemia or diabetes, %	30.3	13.4	<0.001
Raised triglycerides, %	34.2	14.5	<0.001
Reduced HDL cholesterol, %	11.7	9.1	0.379
Elevated high-sensitivity CRP, %	15.2	13.4	0.610
Low physical activity, %	25.5	26.4	0.724
Risky alcohol consumption, %	22.9	5.8	<0.001
Daily smoking, %	41.6	22.9	<0.001
Family history of obesity, %	45.9	45.5	0.502

*Mean and standard deviation; **median and interquartile range.

*Abbreviations: BMI* body mass index, *HDL* high density lipoprotein, *CRP* C-reactive protein.

Prevalence of CVD risk factors was high in the cohort (Table 1). Proportion of adult men having hypertension, metabolic syndrome, hyperglycaemia or type 2 diabetes and raised level of triglycerides was higher than that of women. Risky alcohol consumption and smoking were also more common among men.

Weight, BMI and the skinfold thicknesses in childhood significantly correlated with adult anthropometric measurements (Table 2). The Spearman correlation coefficients were slightly higher in women than in men; however, few differed significantly. The highest correlation coefficients were defined for weight and BMI measurements in childhood and adulthood (in girls r = 0.56 and r = 0.51 respectively; in boys r = 0.45 and r = 0.41 respectively, P < 0.001).

**Table 2:** **The Spearman correlation coefficients between anthropometric measurements in childhood and adulthood**

**Measurements in childhood**	**Measurements in adulthood**		
	**Weight**	**BMI**	**Waist circumference**
**Men**			
Weight	0.45	0.32	0.28^a^
BMI	0.41	0.41	0.30
Triceps skinfold	0.32	0.35	0.28
Subscapular skinfold	0.34	0.34	0.28^a^
**Women**			
Weight	0.56	0.46	0.44
BMI	0.51	0.53	0.46
Triceps skinfold	0.40	0.41	0.40
Subscapular skinfold	0.44	0.47	0.44

All P values <0.001.

^a^statistically significant differences between correlation coefficients of men and women.

*Abbreviations: BMI* body mass index.

BMI gain from childhood to adulthood did not depend on the initial BMI level (Table 3). Namely, mean values of change in BMI were similar in all quintiles of childhood BMI. However, prevalence of adult obesity increased considerably with increasing quintiles. More than a half of children who had the highest BMI (the 5^th^ quintile) were obese as adults. In addition, children whose BMI belonged to the 4^th^ quintile were also at increased risk of adult obesity. From this group, 31.1% of boys and 27.3% of girls became obese by the time of the last survey, as compared to 8.7% of boys and 9.1% of girls with BMI from the 1^st^ quintile.

**Table 3:** **Mean values of change in body mass index (BMI) from childhood to adulthood and prevalence of obesity in adulthood by quintiles of BMI in childhood**

**Quintiles of BMI in childhood**	**Men**			**Women**		
	**Change in BMI**		**Obesity prevalence in adulthood**	**Change in BMI**		**Obesity prevalence in adulthood**
	**Mean**	**SD**	**%**	**Mean**	**SD**	**%**
1st	9,4	3,7	8.7	7,7	4,1	9.1
2nd	9,1	3,7	13.0	7,5	3,1	3.6
3rd	9,7	5,1	23.4	6,8	3,5	10.7
4th	9,6	3,9	31.1	8,2	5,3	27.3
5th	7,5	6,0	50.0	7,8	5,4	56.4
P value	0.534*		<0.001**	0.128*		<0.001**

*P value from analysis of variance; **P value from *χ* ^2^ test.

The logistic regression models unadjusted for BMI gain from childhood to adulthood showed that BMI in childhood predicted development of obesity and central obesity in adults (Table 4). Moreover, childhood BMI was predictive for metabolic syndrome, hyperglycaemia or type 2 diabetes and elevated level of high-sensitivity CRP in adulthood. Importantly, these associations remained significant after adjustment for BMI gain from childhood to adulthood. No relationship was found between childhood BMI and adult arterial hypertension, raised level of triglycerides or reduced level of HDL cholesterol. Gain in BMI was associated with increased odds of all above-mentioned CVD risk factors independently of childhood BMI. Neither childhood BMI, nor gain in BMI were related to total cholesterol and low-density lipoprotein (LDL) cholesterol level (data are not shown).

**Table 4:** **Odds ratios and 95% confidence intervals of cardiovascular risk factors in adulthood according to body mass index (BMI) in childhood and change of BMI from childhood to adulthood**

**Risk factor**	**Models with BMI in childhood**		**Models with BMI in childhood and BMI gain**			
	**OR by BMI in childhood**	**95% CI**	**OR by BMI in childhood**	**95% CI**	**OR by change in BMI**	**95% CI**
Obesity^a^	**1.38**	1.27-1.51	-	-	-	-
Central obesity	**1.23**	1.14-1.33	**2.38**	1.94-2.91	**2.90**	2.36-3.56
Metabolic syndrome	**1.12**	1.05-1.21	**1.21**	1.10-1.32	**1.43**	1.33-1.53
Hyperglycemia or diabetes	**1.13**	1.05-1.21	**1.15**	1.06-1.24	**1.15**	1.10-1.22
Hypertension	1.02	0.95-1.08	1.04	0.97-1.12	**1.26**	1.20-1.34
Raised triglycerides	0.99	0.92-1.07	1.01	0.93-1.09	**1.22**	1.15-1.29
Reduced HDL cholesterol	1.04	0.95-1.14	1.05	0.95-1.16	**1.20**	1.13-1.28
Elevated high-sensitivity CRP	**1.16**	1.07-1.28	**1.19**	1.09-1.31	**1.23**	1.16-1.30

Separate logistic regression models were calculated for every risk factor. Odds ratios were calculated for a one-unit increase in the BMI. All logistic regression models were adjusted for sex, physical activity level, alcohol consumption, smoking and family history of obesity.

^a^Because of complete separation, logistic regression was not fitted for calculation of obesity odds when BMI in childhood and BMI gain were included in the model.

Significant (P < 0.05) values in bold.

*Abbreviations: OR* odds ratio, *CI* confidence interval, *BMI* body mass index, *HDL* high density lipoprotein, *CRP* C-reactive protein.

The skinfold thicknesses in childhood also predicted CVD risk factors in adulthood. The increase in the sum of skinfold thicknesses by 1 standard deviation (SD) was associated with higher odds of obesity and central obesity, metabolic syndrome, hyperglycaemia or type 2 diabetes, and elevated level of high-sensitivity CRP in adulthood (Table 5). The effect of BMI and the skinfold thicknesses on CVD risk factors was not substantially influenced by lifestyle factors (physical activity, alcohol consumption, smoking) and family history of obesity.

**Table 5:** **Odds ratios and 95% confidence intervals of cardiovascular risk factors in adulthood according to the sum of subscapular and triceps skinfold thicknesses in childhood**

**Risk factor**	**Models unadjusted for BMI gain**		**Models adjusted for BMI gain**	
	**OR**	**95% CI**	**OR**	**95% CI**
Obesity	**2.20**	1.72-2.83	**3.10**	1.20-8.08
Central obesity	**1.77**	1.36-2.29	**6.14**	3.84-9.84
Metabolic syndrome	**1.29**	1.05-1.60	**1.52**	1.16-2.00
Hyperglycemia or diabetes	**1.43**	1.15-1.78	**1.52**	1.21-1.92
Hypertension	1.01	0.82-1.22	1.06	0.85-1.32
Raised triglycerides	1.00	0.79-1.25	1.03	0.80-1.32
Reduced HDL cholesterol	1.08	0.81-1.43	1.10	0.80-1.50
Elevated high-sensitivity CRP	**1.59**	1.26-2.00	**1.78**	1.34-2.33

Separate logistic regression models were calculated for every risk factor. OR was calculated for a 1-SD increase in the sum of skinfold thicknesses. All logistic regression models were adjusted for sex, physical activity level, alcohol consumption, smoking and family history of obesity. Significant (P < 0.05) values in bold.

*Abbreviations: OR* odds ratio, *CI* confidence interval, *BMI* body mass index, *HDL* high density lipoprotein, *CRP* C-reactive protein.

## Discussion

Our study has shown that anthropometric measurements in childhood strongly correlated with values measured in adulthood. The risk of adult obesity increased with a rise in childhood BMI and skinfold thickness. Moreover, anthropometric measurements in childhood were associated with the risk of metabolic syndrome, hyperglycaemia or type 2 diabetes, and elevated level of high-sensitivity CRP in adults, irrespectively of BMI gain from childhood to adulthood.

Metabolic risk factors related to cardiovascular health are highly prevalent in Lithuanian population [22]. Such adverse cardiovascular risk profile is a major contributor to high CVD mortality, which is one of the highest in Europe [16]. Our findings suggest that the impact of adiposity on cardiovascular risk begins early in life. Thus the implementation of obesity prevention and control measures in childhood might be one of the strategies for CVD risk reduction in Lithuania.

Previous prospective studies found significant and strong tracking between childhood and adulthood BMI values [4-7,23]. In the Young Finns Study, Spearman correlation coefficients for 27-year tracking of BMI of 12-year-old children were similar to those determined in our cohort (r = 0.59 for men and r = 0.45 for women) [7]. Some researchers reported associations between weight status in childhood and adulthood being stronger for girls than for boys [4,7,23]. However, opposite findings were also published [24,25]. We did not find any substantial gender differences in the associations between anthropometric measurements in childhood and adulthood.

Studies that analysed the risk of becoming overweight by strata of childhood weight revealed that persistence of overweight was greater with increasing childhood BMI [4,23]. A systematic review showed that the risk of overweight children to become overweight adults was at least twice as high as compared with normal-weight children [5]. Moreover, Field et al. demonstrated that children in the upper end of the healthy weight range (50^th^ to 84^th^ percentile) also were at an elevated risk of becoming overweight in adulthood [4]. Similar to this study, our data indicated that almost one third of children with BMI from the 4^th^ quintile became obese as adults. These findings suggest that prevention of adult obesity should be targeted not only at overweight children, but also at children in the upper part of normal weight range, especially in the context of contemporary obesogenic environment.

Epidemiological studies proved that overweight in childhood predicts the development of CVD in adulthood [26-28]. In our study, childhood BMI was associated with CVD risk factors such as obesity, metabolic syndrome, and hyperglycaemia. Although some authors questioned the causality of the association of CRP with metabolic disorders and related chronic diseases [29], we found that childhood BMI was associated with elevated level of high-sensitivity CRP in adults. Meanwhile, childhood BMI was not predictive of adult hypertension or dyslipidaemias. Our results are consistent with the findings reported by Li L et al. who also established that childhood BMI was a weak predictor of adult hypertension and dyslipidaemias [6]. The pooled analysis of three British birth cohorts also showed that overweight in childhood and adolescence was associated with an increased risk of type 2 diabetes, while exposure to overweight in early life did not influence adult BP [15]. On the contrary, some longitudinal cohort studies found statistically significant associations of overweight in childhood with hypertension, high level of LDL cholesterol and triglycerides as well as low level of HDL cholesterol in adulthood [14].

Several studies examined the association between childhood BMI and metabolic syndrome in adults [30,31]. They reported that a rise in childhood BMI increased the odds of developing the metabolic syndrome. However, Lloyd LJ et al. found little evidence that childhood obesity is an independent risk factor for adult metabolic syndrome [32]. The authors argued that the associations might reflect the tracking of BMI because the majority of the studies did not adjust for adult BMI. In the present study, BMI gain was included into a logistic regression model; however, the adjustment did not diminish the effect of childhood BMI for the prediction of metabolic syndrome.

Compared to BMI, skinfold thicknesses were more strongly associated with body fatness; therefore, it may be assumed that skinfold thicknesses would better predict CVD risk than BMI [33]. However, limited evidence exists on the effect of childhood skinfold thicknesses for the prediction of CVD risk in adults. In our study, the sum of subscapular and triceps skinfold thicknesses in childhood was associated with odds of obesity and central obesity, metabolic syndrome, hyperglycaemia or type 2 diabetes, and elevated high-sensitivity CRP in adulthood. Other researchers also found that CVD risk factors were related to both skinfold thicknesses and BMI; however, differences in the magnitude of these associations were small [34,35]. Freedman DS et al. demonstrated that BMI was at least as accurate as skinfold thicknesses in identifying metabolic risk in adults [35]. As compared to height and weight, a chance of errors is higher for skinfold thickness measurements; therefore, BMI is assumed to be more appropriate for assessment of weight status in childhood.

Our data demonstrated that not only childhood BMI, but also BMI gain from childhood were associated with adverse levels of cardiovascular risk factors in adulthood. Prospective studies carried out in UK and USA also found that BMI gain substantially increases CVD risk [36,37]. In adults of the same BMI, the risk of metabolic disorders differed depending on BMI gain from childhood to adulthood. Indeed, the individuals with persistent overweight from childhood to adulthood were at a higher cardiovascular risk compared to those with adult-onset obesity [14,15]. On the contrary, the individuals whose adiposity decreased (relative to age and sex standards) had more favourable cardiovascular risk profiles [14,37]. These data emphasize that excessive weight gain should be prevented at any stage of life in order to reduce the risk of CVD.

The strength of the present study is the randomly selected cohort followed 35 years since childhood. All anthropometric measurements were carefully taken using standardized methodology in childhood and adulthood. A further strength includes the adjustment of odds of CVD risk factors for major life-style characteristics. The study has several limitations. During the 35-year follow-up, the loss of participants was quite substantial. A high rate of emigration from Lithuania over the last decades was one of the reasons for non-participation in the follow-up survey. Although no differences in baseline measurements were identified between participants and non-participants, the trends in BMI and CVD risk factors during the life course might be different in those groups. The loss to follow-up might have resulted in selection bias if non-response was associated with BMI gain and incidence of CVD risk factors. Our findings might not be generalized to Lithuanian population because baseline survey was carried out only in Kaunas city. However, a substantial proportion of cohort has spread all over the country during follow-up period. There is no reason to believe that the association between anthropometric measurements and CVD risk in our cohort is different from whole population. Finally, we were not able to adjust odds ratios for the changes in lifestyle factors due to lack of information about these factors in the baseline survey.

## Conclusions

Childhood BMI was associated with risk of adult obesity, metabolic syndrome, hyperglycaemia or diabetes, and elevated high-sensitivity CRP, while risk of hypertension, raised triglycerides and reduced HDL cholesterol was predominantly affected by BMI gain from childhood to adulthood. The associations of the skinfold thicknesses with cardiovascular risk factors were similar to those of BMI. From a public health perspective, our study emphasizes the importance of primary prevention of overweight from early childhood with continuation of health promotion activities throughout the course of life.

## Abbreviations

BMI: Body mass index
BP: Blood pressure
CHD: Coronary heart disease
CRP: C-reactive protein
CVD: Cardiovascular disease
HDL: High-density lipoproteins
IOTF: International Obesity Task Force
LDL: Low-density lipoproteins
OR: Odds ratio
SD: Standard deviation
